# Early Detection of Rhabdomyolysis-Induced Acute Kidney Injury through Machine Learning Approaches

**DOI:** 10.22037/aaem.v9i1.1059

**Published:** 2021-03-25

**Authors:** Pooria Poorsarvi Tehrani, Hamed Malek

**Affiliations:** 1 Faculty of Computer Science and Engineering, Shahid Beheshti University, Tehran, Iran.

**Keywords:** Acute Kidney Injury, Clinical Decision Rules, Machine Learning, Neural Networks, Computer, Decision Making

## Abstract

**Introduction:**

Rhabdomyolysis-induced acute kidney injury (AKI) is one of the most common complications of catastrophic incidents, especially earthquakes. Early detection of AKI can reduce the burden of the disease. In this paper, data collected from the Bam earthquake was used to find a suitable model that can be used in prediction of AKI in the early stages of the disaster.

**Methods:**

Models used in this paper utilized many inputs, which were extracted from the previously published dataset, but depending on the employed method, other inputs have also been considered. This work has been done in two parts. In the first part, the models were constructed from a smaller set of records, which included all of the required fields and in the second part; the main purpose was to find a way to replace the missing data, as data are mostly incomplete in catastrophic events. The data used belonged to the victims of the Bam earthquake, who were admitted to different hospitals. These data were collected on the first day of the incident via questionnaires that were provided by the Iranian Society of Nephrology, in collaboration with the International Society of Nephrology (ISN).

**Results:**

Overall, neural networks have more robust results and given that they can be trained on more data to gain better accuracy, and gain more generalization, they show promising results. Overall, the best specificity that was achieved on testing almost all of the records was 99.24% and the best sensitivity that was achieved in testing almost all of the records was 94.44%.

**Conclusion:**

We introduced several machine learning-based methods for predicting rhabdomyolysis-induced AKI on the third day after a catastrophic incident. The introduced models show higher accuracy compared to previous works performed on the Bam earthquake dataset.

## Introduction

Rhabdomyolysis-induced acute kidney injury (AKI) is one of the main medical complications of catastrophes, and is the second leading cause of death in traumatic injuries. After being pulled out from the rubble, dehydration and circulatory defects may occur, which can eventually lead to AKI (1). 

A lot of work has been done for predicting AKI, in most of which Electronic Health Records (EHR) and a logistic regression to predict the state of a patient (2-5) or a linear regression to predict another value are used, so that they can predict the state of the patient using an estimated value (6). 

Even though a great amount of research has been done in this field, most of the models proposed in the past have a trade-off on sensitivity and specificity. After training these models for some time they either gain a relatively very high sensitivity or very high specificity. Most of the models are also trained on EHRs with lots of columns and very little missing data, and although they are useful in training a machine learning model that is not the case in catastrophic events, in which most of the time the information is only partially available. 

Most of these models use linear or logistic regression and even though these models are easier to implement in a Decision Support System (DSS), they might fail to capture some of the non-linearities in the data. The proposed models in this paper will try to use non-linear models to reach a better accuracy and also to reach a sensible level of invariance to the loss of information.

## Methods

The dataset used in this study is from Najafi et al. (6), which was collected by the Iranian Society of Nephrology, in collaboration with the International Society of Nephrology (ISN) on the first day of the Bam earthquake. In that work, a questionnaire was developed and sent to all hospitals that were involved in the treatment of patients. In addition to basic demographic data, some biochemical factors were collected, which include serum creatinine, creatine phosphokinase (CPK), lactate dehydrogenase (LDH), serum glutamic-oxaloacetic transaminase (SGOT), uric acid, calcium (Ca), phosphorus (P), sodium (Na), potassium (K), white blood cell count (WBC) and platelet count (Plt). The details of the protocol including the eligibility criteria have been presented in their article (6).

Due to sparsity of data, the procedure of building the final models was performed in two parts. In the first part, we tried to construct the models only based on the parts of the Bam dataset that were employed in the work of Najafi et al. (6) and in the second part of the paper, a method that can be used in order to use all of the Bam dataset is proposed. So, the models were built in the following order:

- **Models built from records with available fields**

- Regression plus classification neural networks (RC_NN).

In the first stage, records that have all of the desired fields (CPK, LDH, Potassium, Uric acid, and creatinine on the 3^rd^ day) are employed to build a prediction model for creatinine on the 3^rd^ day, and in the second stage, a classification neural network is used to predict AKI occurrence.

- Full Neural Network Model (FNNM).

Using a neural network instead of a threshold to predict whether or not someone is diagnosed with AKI. This is the second part of our RC_NN.

- Using Genetic Programming to predict whether or not someone is diagnosed with AKI.

- **Models built from all records**

- Using two neural networks with different neural network architectures.
- Using a Support Vector Machine (SVM) to predict whether or not someone is diagnosed with AKI.
- Using a Random Forest, to predict whether or not someone is diagnosed with AKI.
- Using the last four models to make an ensemble model, to predict whether or not someone is diagnosed with AKI.

For the implementation of the algorithms, all of the neural network models were made using Keras (7) with the back end of Tensorflow (8) and the random forest and the support vector machine models were made using scikit-learn. The model that uses genetic programming was made using GP Learn, a python library.

Models on partial data from the Bam earthquake

In this section, in order to build a model, biochemical factors used in the work of Najafi et al. (6) were employed and any record that had missing information was removed. The biochemical factors used in this part are: CPK, LDH, Potassium, and Uric acid. The aforementioned factors are used either to predict the state of a patient directly or to predict the value of creatinine on the third day, so that it can later be used in order to predict the state of a patient.

Predicting using neural networks

**Figure 1:**
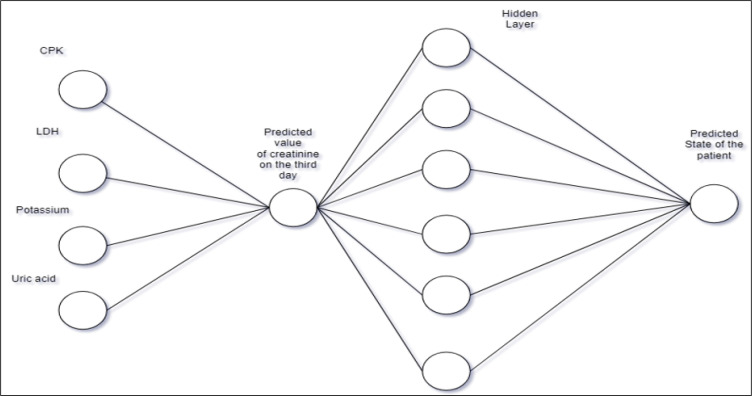
The architecture of the first model from available data (FC_NN). CPK: Creatine phosphokinase; LDH: lactate dehydrogenase

In the first model, two different parts, both of which are neural networks, were employed. The first one is used to predict the normalized value of creatinine on the third day; the second neural network is used to predict whether or not someone should be diagnosed with AKI considering the predicted value of creatinine on the third day. The Architecture of the models is presented in Figure 1. 

Using a neural network instead of a threshold

In the second model, a neural network was used instead of using a threshold on the predicted value of creatinine on the third day. This model had more accuracy on the 553 rows that had the value of creatinine on the third day than the single threshold that was introduced in the work of Najafi et al. (6).

Using genetic programming to predict the state of patients

Genetic programming is a method that is inspired by biological evolution. First, different individuals or candidates are created. Then, these individuals are combined and at times mutated so that they can change over time. These individuals are also evaluated at each step so that the ones that have better performance (fitness), which is their accuracy on the training dataset, can continue to the future generations. This model constructs a tree made up of functions and variables and constants. The functions that were considered are the following: add, sub, multiplication, division, max, log, sqrt, and abs. The range of constants was from -1.5 to +1.5. The variables were also the same biochemical factors that were used in 3.1: CPK, LDH, Potassium (K), and Uric acid. 

Models on all data from the Bam dataset

One of the main difficulties for algorithms that are introduced in ‎2.1 is that data gathered from catastrophes usually have lots of missing information and the aforementioned algorithms in ‎2.1 do not work well with missing data, especially if all of these missing data were to be replaced with the mean of the dataset, as it is usually done. Here we introduce another method for replacing these missing data that works better than simply replacing them with the mean of dataset. For the models built in this section, a broader range of biochemical factors were considered. These factors are: creatinine on the first day, SGOT, phosphate, CPK, LDH, WBC, plt, Uric Acid, Na, K, Ca, Age, Gender, and creatinine on the third day. The last factor is only used to train a model to try and predict the value of the creatinine on the third day and after that, all values of creatinine are dropped. 

Missing values

For all factors except age and creatinine on the 3^rd^ day, data collected from previous studies were employed and missing data were replaced with previously known values for healthy humans. The aforementioned values are listed in Table 1. The missing values in these columns are replaced accordingly so that models will not be very dependent on the mean of the data they are being trained on and if some column is missing, they will try to act in a way that the aforementioned factor is fine and the model has to diagnose the patient based on other factors. It should also be mentioned that for other values that were not mentioned, we did use the mean of the training data set. 

For prediction of age, a neural network was used. There was also missing data regarding gender, but most of the time this information is available, even in catastrophes. So, the information of patients whose gender was unknown were dropped. The number of these patients was less than 40 in 1440 patients.

In order to predict the value of creatinine on the third day, another neural network was used, whether or not the information was available, because in real situations this information is usually not available and after the model was trained on the values of the patients whose information was available, all information of creatinine on the third day was dropped and then that value was predicted for everyone and then the next parts of the study were proceeded with. 

Neural network 

A multi-layer perceptron neural network was constructed to predict AKI. This neural network is trained to penalize wrong outputs for patients who are diagnosed with AKI. The aforementioned training procedure might result in overfitting, so a regularization algorithm called dropout was also used during the training. Dropout can prevent overfitting as discussed in the work of Srivastava et al. (9). 

The loss function in this model is very similar to cross entropy for binary classification:

$\mathrm{loss}\left(y,y^{j}\right)=-\left(w_{1}y\log\left(y^{j}\right)+w_{2}\left(1-y\right)\log\left(1-y^{j}\right)\right),$

where the values of $w_{1}$ and $w_{2}$ are equal to 2.8 and 0.07, respectively. 

Random Forest

Decision trees are often used in medical applications because they can easily be implemented into DSSs. These models try to choose one biochemical factor at each step to separate the data set, and this procedure is continued until the data set is separated into the target classes. In a random forest, a group of decision trees are trained in which a grid search is used to select the best number of estimators, or decision trees. The values that were considered for the number of estimators were selected from 1 to 2000.

Support Vector Machine

Support Vector Machines (SVMs) are used to find the best hyperplane to separate data in order to classify them. This hyperplane is found in a manner that has maximum classification margin with data. In order to model the non-linear relations, a kernel is used to transform the space of our data to higher dimensions. Grid search was also used for support vector machine part of the work, where the values of c were selected from [0.1, 0.5, 1, 5, 10, 50, 100, 500, 1000,5000, 10000] and the values for gamma from: [1, 0.5,0.1, 0.05,0.01, 0.005, 0.001, 0.0005, 0.0001]. In order to better approximate the non-linearities, RBF kernel was used (10).

Ensembled Method

Different versions of ensemble were tested, and in the end, the outputs of the last four models were entered into a logistic regression unit in order to have the final prediction.

Cross Validation

All of the models were validated using k-folds, in some cases, 5 was chosen as k and for others, 10 was chosen. Using k-fold validation enables us to assess how the trained model is able to generalize on an independent dataset. This would help to flag problems like over-fitting or selection bias (11, 12).

## Results

The sensitivity and specificity of the implemented models are provided in Table 2. In the first step, the model introduced in ‎2.1.1 was used to train on records that had all of the following biochemical factors: CPK, LDH, Potassium, Uric acid and the value of creatinine on the third day. This data is used to learn how to predict the value of creatinine on the third day. After this training procedure, this model is trained and tested on two different parts of the datasets, the first one is the same as the one used to learn how to predict the value of creatinine on the third day, and the second one is the part of the dataset, which has the biochemical factors: CPK, LDH, Potassium and Uric acid. 

As you can see, the model’s accuracy will decrease a lot in the second part, because a lot of the data has been removed and the first part of this model has only been trained on 162 records so it has a generalization problem. This problem is solved in ‎2.2 when almost all of the data is used and the accuracy is higher.

In the Neural Network model, a procedure is proposed to predict the state of a patient using the information from the value of creatinine on the third day. In comparison to the previous work of Najafi et al. (6), which used a threshold for diagnosing the state of a patient, in the proposed method, a neural network is employed. 

This model is trained on the real values of creatinine and only tries to predict the state of a patient and does not regress the value of creatinine.

The genetic programming (GP) model introduced in ‎2.1.3 uses the part of the dataset, which includes data about the following biochemical factors: CPK, LDH, Potassium, and Uric acid. It has 239 rows with 4 columns. The best formula that was derived from this model was the following: 

Output = log K* Max[0.613, Max(K, UricAcid)*LDH]

In models built on all data samples, k-fold was used for validation, with k=10. As mentioned before, all of the collected information about the value of creatinine on the third day was dropped after the models learned how to predict it themselves. 

## Discussion

In this paper, different machine learning models were introduced for predicting AKI in catastrophic events. In comparison to the previous work of Najafi et al. (6) on this dataset, the models yielded higher sensitivity and specificity for prediction of AKI on day 3. 

The models show their strength in different scenarios. Random forests are made up from decision trees, which are easier to interpret in comparison with neural networks that act like black boxes, but as evident in the results, neural networks perform better than random forests. Support vector machines are also easier to implement into DSS but have more performance issues. Overall, the best specificity that was achieved on testing almost all of the records was 99.24% and the best sensitivity that was achieved in testing almost all of the records was 94.44%. Overall, neural networks have more robust results and given that they can be trained on more data to gain better accuracy, and gain more generalization, they show promising results.

**Table 1:** Replacement of missing values with normal values for healthy humans

**Variable**	**Value for males**	**Value for females**
CPK (IU/L)	57.5	40
Sodium (mEq/L)	140	140
Potassium (mEq/L)	4.75	4.75
Calcium (mEq/L)	9.5083	9.475
LDH (IU/L)	219	219
Uric acid (mg/dL)	5.2	4.2
PLT (/*mm*^3^)	350000	350000
WBC (/*mm*^3^)	7400	7400

CPK: Creatine phosphokinase; LDH: lactate dehydrogenase; PLT: platelet; WBC: white blood cell.

**Table 2:** Specificity and Sensitivity of different models (95% confidence interval)

**Partial Data fields**	**Model** **s**	**Phase**	**Specificity**	**Sensitivity**
	RC-NN on Part1	Train	99.91 (97.95-99.93)	99.58 (97.62-100)
		Test	99.37 (97.41-100)	100.00 (98.04-100)
	RC-NN on Part2	Train	99.27 (96.92-100)	92.49 (90.13-94.84)
		Test	99.51(97.16-100)	89.28 (86.93-91.63)
	Neural Network Model	Train	99.42 (97.26-100)	97.04 (94.88-99.19)
		Test	99.37 (97.21-100)	96.57 (94.41-98.72)
	Genetic Programming	Train	99.51 (96.76-100)	90.53 (87.77-93.25)
		Test	98.00 (95.26-99.65)	91.47 (88.72-94.21)
**All Data fields**	Neural Network Model	Train	93.54 (91.77-95.30)	100.00 (98.23-100)
		Test	93.04 (91.27-94.80)	94.44 (92.67-96.21)
	Random Forest	Train	100.00 (97.35-100)	100.00 (97.15-100)
		Test	99.24 (96.59-100)	90.24 (87.40-93.08)
	Support Vector Machine	Train	99.84 (97.64-100)	89.64 (87.44-91.83)
		Test	99.69 (97.49-100)	83.12 (80.92-85.31)
	Random Forest	Train	99.98 (97.59-100)	100 (97.61-100)
		Test	99.47 (96.82-100)	89.20 (86.55-91.84)
	Support Vector Machine	Train	99.84 (97.64-100)	90.49 (88.29-92.68)
		Test	99.62 (97.42-100)	84.13 (81.93-86.32)
	Ensembled	Train	99.89 (98.32-100)	96.58 (95.01-98.15)
		Test	99.54 (97.97-100)	90.21 (88.64-91.78)

## Limitations:

Some of the best results achieved in this work are from non-linear models like neural networks. When complex non-linear models, relative to the amount of data available. are employed, the potential for overfitting of the model to the training data is high. Although various methods have been used to solve the problem of overfitting, it seems that linear models can still be a better option, especially in situations where explainability is important.

## Conclusion:

In this article, we introduced several machine learning-based methods for predicting AKI on the third day after a catastrophic incident. The introduced models show higher accuracy compared to previous works performed on the Bam earthquake dataset. In the proposed models, an attempt was made to maintain the generalizability of the models by considering the missing data and replacing them with appropriate values, as well as using various regularization and validation methods such as dropout and cross-validation. Due to the variety of models, it is possible to use each of these models in different conditions and for different applications.

## Support

Nothing to declare
